# FROM MISFIT TO FIT: THE IMPACT OF NURSING PRACTICE ON COMPLEX NURSING INTERVENTION DEVELOPMENT

**DOI:** 10.1093/geroni/igad104.2461

**Published:** 2023-12-21

**Authors:** Debbie ten Cate, Marieke Schuurmans, Lisette Schoonhoven, Roelof Ettema

**Affiliations:** Utrecht University of Applied Sciences, Utrecht, Utrecht, Netherlands; Dutch Healthcare Authority, Utrecht, Utrecht, Netherlands; University Medical Center Utrecht, Utrecht University, Utrecht, Utrecht, Netherlands; Utrecht University of Applied Sciences, Utrecht, Utrecht, Netherlands

## Abstract

Nurses have a key role in providing nutritional care to older adults to prevent and treat malnutrition, and stimulate health and well-being. However, evidence for nursing activities regarding nutritional care is often lacking. Therefore, intervention development is necessary. From earlier studies, an evidence-based nutritional intervention carried out by nurses appeared the best solution. The aim of this study was to outline the steps taken to develop a complex nursing intervention to prevent and treat malnutrition in older adults and the challenges faced during this stage. Following the phase of intervention development of the Medical Research Council (MRC) Framework, a systematic review, a questionnaire survey, semi-structured interviews, focus groups and participant observation were carried out. The purpose was to gather information about current nursing practice and context, the needs of future users and providers, and gain insight into the problem. The studies showed that nurses had moderate knowledge of (mal)nutrition. They gave nutritional care low prioritization during daily nursing activities. These results provided insight that the intended nursing nutritional intervention would most likely lead to a misfit with the context. To fit the intervention properly into nursing practice, it was decided to develop an educational intervention targeting nurses instead of a nutritional intervention carried out by nurses. Building proofs in context leads to challenges but is essential to prevent a misfit between complex nursing interventions and nursing practice. As an element of rigorous intervention development following systematic steps, it increases the chance of successful implementation.

